# State of the art technologies to explore long non‐coding RNAs in cancer

**DOI:** 10.1111/jcmm.13238

**Published:** 2017-06-19

**Authors:** Saeede Salehi, Mohammad Naser Taheri, Negar Azarpira, Abdolhossein Zare, Abbas Behzad‐Behbahani

**Affiliations:** ^1^ Diagnostic Laboratory Sciences and Technology Research Center School of Paramedical Sciences Shiraz University of Medical Sciences Shiraz Iran; ^2^ Department of Medical Biotechnology School of Paramedical Sciences Shiraz University of Medical Sciences Shiraz Iran; ^3^ Student Research Committee Shiraz University of Medical Sciences Shiraz Iran; ^4^ Transplant Research Center Namazi Hospital Shiraz University of Medical Sciences Shiraz Iran

**Keywords:** long non‐coding RNAs (lncRNAs), cancer, experimental techniques, interaction networks, lncRNA function

## Abstract

Long non‐coding RNAs (lncRNAs) comprise a vast repertoire of RNAs playing a wide variety of crucial roles in tissue physiology in a cell‐specific manner. Despite being engaged in myriads of regulatory mechanisms, many lncRNAs have still remained to be assigned any functions. A constellation of experimental techniques including single‐molecule RNA *in situ* hybridization (sm‐RNA FISH), cross‐linking and immunoprecipitation (CLIP), RNA interference (RNAi), Clustered regularly interspaced short palindromic repeats (CRISPR) and so forth has been employed to shed light on lncRNA cellular localization, structure, interaction networks and functions. Here, we review these and other experimental approaches in common use for identification and characterization of lncRNAs, particularly those involved in different types of cancer, with focus on merits and demerits of each technique.

**Table:** 

• Introduction
• LncRNAs: the emerging master regulators of gene expression and cell fate
• LncRNAs: key players in cancer biology and diagnosis
• Methods used to localize lncRNAs inside the cell
– Single‐molecule RNA *in situ* hybridization (sm‐RNA FISH)
– RNA Mimics of GFP and RNA visualization
– Clustered regularly interspaced short palindromic repeats (CRISPR) RNA Tracking
• Methods used to predict lncRNA structure
– Selective 2′‐hydroxyl analysed by primer extension (SHAPE)
– Parallel analysis of RNA structure (PARS)
– Domain‐specific chromatin isolation by RNA purification (dChIRP)
• Methods utilized for determination of lncRNA function
– Knockout techniques
Clustered regularly interspaced short palindromic repeats (CRISPR) Cas technology
– Knock‐down techniques
RNA interference (RNAi)
CRISPR Interference (CRISPRi)
Combined knock‐down and localization analysis of non‐coding RNAs (c‐KLAN)
Antisense oligonucleotides (ASOs)
• Methods used for mapping lncRNA interactions
– Mapping lncRNA–DNA interactions
ChIRP, CHART and RAP as RNA‐centric methods to map lncRNA genomic binding sites
– Mapping lncRNA–protein interactions by protein pull‐down
RNA immunoprecipitation (RIP)
Cross‐linking and immunoprecipitation (CLIP)
• Next‐generation sequencing‐based methods for global investigation of lncRNAs
– LIGation of interacting RNA followed by high‐throughput sequencing (LIGR‐seq)
– Inosine Chemical Erasing Sequencing (ICE‐seq)
– Global mapping of lncRNA–protein interactions based on RNA modification and RNA‐seq
– Global run‐on assay sequencing (Gro‐seq)
– 5′‐bromo‐uridine immunoprecipitation chase–deep sequencing (BRIC‐seq)
– Fluorescent *in situ* sequencing (FISSEQ)
• Conclusion

## Introduction

In the post‐genomic era, the ‘junk’ moniker is giving way to the understanding that most of the genome is fully functional 1. We are facing an expanding RNA universe filled with transcripts of unknown functions (TUFs) 1. The catalogue of non‐coding RNAs (ncRNAs) is skyrocketing in an ever‐increasing way and the part of the human genome (nearly two‐thirds) transcribed into non‐coding RNAs vastly outweighs the part apportioned to protein‐coding genes 1. Among recently highlighted ncRNAs are lncRNAs that surpass 200 base pairs in size 1. According to a current GENCODE release, 9640 loci encoding 15512 lncRNA transcripts have been identified by now 2. Parts of the human genome undergoing active transcription can be distinguished from other parts by specific signatures 3. Important among such signatures are a stretch of trimethylation of histone at the lysine in position 4 (H3K4me3) associated with the promoter areas, followed by another stretch of trimethylation of histone at the lysine in position 36 (H3K36me3) spanning the whole transcribed region 3, 4. Among transcripts encoded by these regions are long non‐coding RNAs 5.

Most lncRNAs are transcribed by RNA polymerase II 1, and similar to protein‐coding RNAs, can be subjected to post‐transcriptional modifications, including 5′‐end capping, 3′‐end polyadenylation, splicing 6, 7, 8, 9, 10, 11 N^6^ adenosine methylation (m^6^A) 12 and A‐to‐I editing 13. Due to not having a comprehensive knowledge of their function, LncRNAs are categorized based on their location relative to the protein‐coding genes as: sense (which are transcribed from sense strand of protein‐coding genes and overlap one or more exons and introns), antisense (which are transcribed from the antisense strand of protein‐coding genes), bidirectional (which are located 1 kb away from promoters of protein‐coding genes in the opposite direction), intronic (which are located between exons of protein‐coding genes) and intergenic (which are located between two protein‐coding genes) 5, 7, 14, 15. On the other hand, other lncRNAs including natural antisense transcripts (NATs) 16, circular RNAs (cRNAs) 17, 18, enhancer ncRNAs (eRNAs) 19, 20, 21, transcribed pseudogenes 22 and telomerase RNA component (TERC) 23 have been also identified.

## LncRNAs: the emerging master regulators of gene expression and cell fate

LncRNAs play a diversity of regulatory functions due to their modular structures and RNA–RNA or RNA–DNA base pairing ability 5. Their modularity enables them to act as scaffolds, providing docking sites for proteins that function together in a given biological process 5.

**Figure 1:**
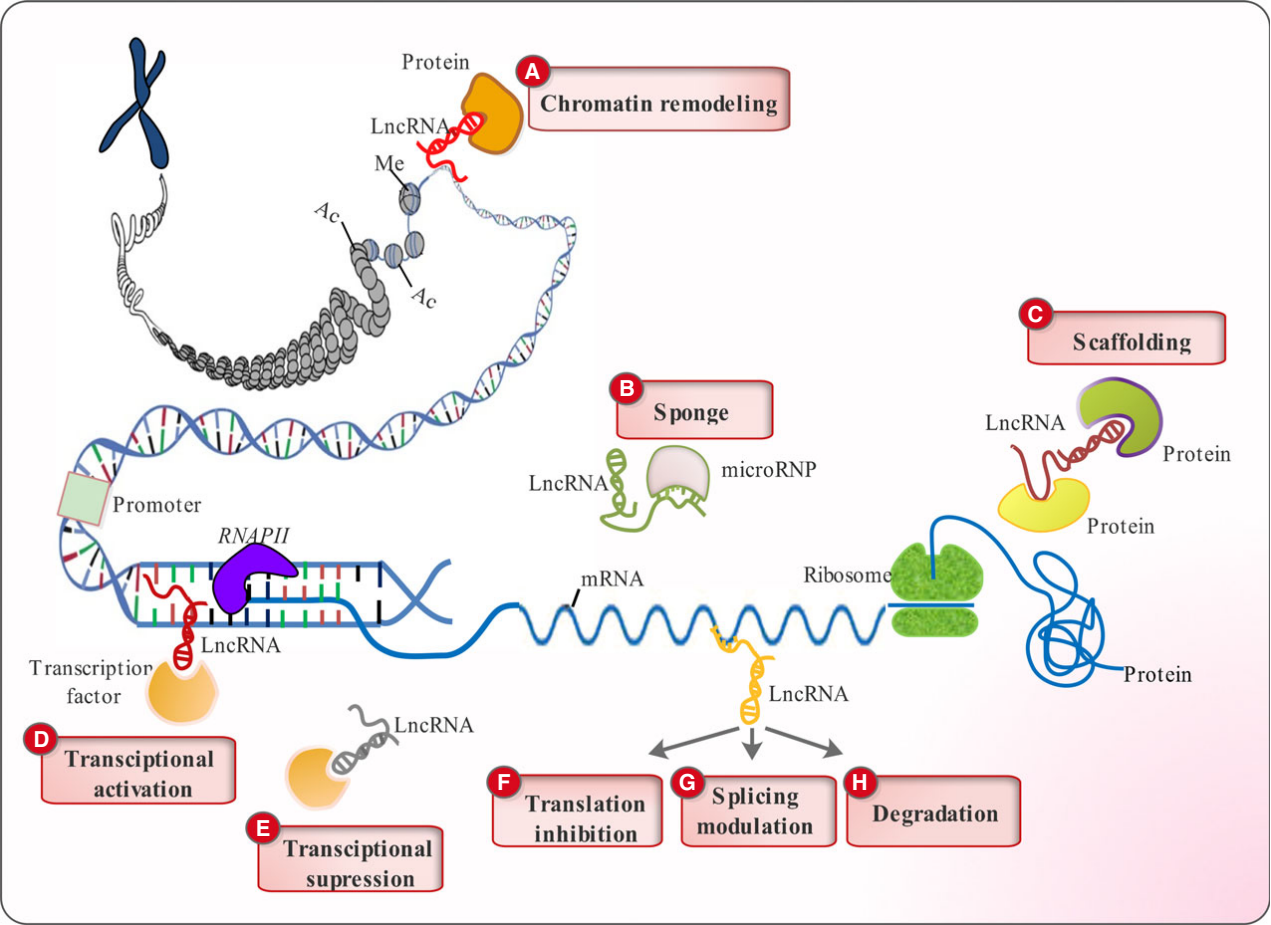
Classification of lncRNA functions. (**A**) LncRNAs can recruit different protein components of the chromatin remodelling complex to change the chromatin organizational patterns. (**B**) They can act as ‘sponges’ by base pairing with their complementary miRNAs and reducing their effects. (**C**) LncRNAs can play scaffolding roles by providing docking sites for proteins that function together in the same biological pathway. (**D**) They activate transcription of certain genes by guiding transcription factors to their promoters. (**E**) LncRNAs are capable of suppressing transcription by sequestering transcription factors and keeping them away from their promoters. They can modulate mRNA functioning through base pairing with them and (**F**) inhibiting their translation (**G**) altering their splicing patterns and (**H**) subjecting them to degradative pathways.

By combining their modularity and base‐pairing ability, lncRNAs act as guiding molecules, directing proteins to other RNA or DNA molecules 24. For instance, the lncRNA HOTAIR was illustrated to modify the epigenetic state of the *HOXD* gene by binding two enzymes (PRC2 and LSD1) involved in chromatin modification 24. In a series of HOTAIR deletion studies, the binding motifs for PRC2 and LSD1 were mapped to nucleotides 1‐300 and 1500‐2146, revealing two structural modules that bind and recruit the two proteins to the *HOXD* gene, where PRC2 deposits the H3K27me3 modification needed for repression and LSD1–CoREST–REST erases the H3K4me2 modification involved in activation 24. As ‘sponges’, lncRNAs hybridize to miRNAs and prevent them from exerting their effects 25, 26. The lncRNA, Cancer Susceptibility Candidate 2 (CASC2), sponges miR‐18a and prevents its down‐regulatory effect on PIAS3 which is an inhibitor of STAT pathway 25. In another case, lncRNAs regulate mRNA functioning by interacting with them and blocking their translation or manipulating their splicing patterns 27. Generally, the list of lncRNA regulatory functions at the molecular level includes chromatin modification 28, genomic imprinting 28, chromosomal dosage compensation 29 and alternative splicing 30 (Fig. 1). From a cellular perspective, lncRNAs play central regulatory roles in cell differentiation 1, cell cycle regulation 31, programming of stem cells 32 and apoptosis 33.

## LncRNAs: key players in cancer biology and diagnosis

LncRNAs, now being emerged as master regulators of gene expression, have been shown to be involved in a variety of malignancies 34. They can be an integral part at the frontline of research in cancer arena, as many of them are involved in tumour progression and aggressiveness by epigenetically silencing tumour suppressors 35. HOTAIR, for instance, stimulates breast cancer metastasis by targeting PRC2 and LSD1‐CoREST complexes to metastasis suppressor genes (such as *PCDH10, PCDHB5* and *JAM2*) 36. From another perspective, some lncRNAs serving as tumour suppressors, contribute to cancer progression, when down‐regulated 37 Supplementary Table 1.

In addition, lncRNAs have been proven as important diagnostic and prognostic biomarkers of cancer and other disorders 38. They have tissue‐ and cancer‐specific expression profiles and can be easily detected in blood and urine samples 38. For instance, PCA3 is a prostate specific lncRNA, overexpressed in prostate cancer. Diagnostic tests have been developed for detection of PCA3 in urine samples as a biomarker for non‐invasive diagnosis of prostate cancer 38, 39.

Thanks to advances in increasing the depth of RNA sequencing techniques, thousands of novel lncRNAs have been discovered in recent years. However, the task of characterizing newly discovered lncRNAs is an arduous one, contingent on the power of technologies at our disposal.

In this review, we strive to shed light on the ever‐expanding toolkit of techniques employed in lncRNA research. The methods are classified based on methodology as techniques used to visualize lncRNAs, determine their structure, map their interactions with other cellular components and elucidate their functions. Each method is described briefly, with their attributed pros and cons being discussed and summarized.

## Methods used to localize lncRNAs inside the cell

Most of the genomic sequence is transcribed, and the majority of such transcripts do not code for proteins 40. Unlike mRNAs whose sequence is a valuable source of insight into their functions, the sequence of lncRNAs usually do not provide much information about their function. Therefore, shedding light on the subcellular localization of lncRNAs can be a first step in revealing their functions in cells and malfunctioning in disease conditions including cancer 41. Here, we review cutting‐edge techniques, which can be employed in determining the localization of lncRNAs.

### Single‐molecule RNA *in situ* hybridization (sm‐RNA FISH)

As with other molecules, our knowledge of the subcellular localization of lncRNAs opens up new vistas to their potential cellular roles 42. RNA FISH has been utilized for many years to localize RNA molecules inside the cells 43. All FISH techniques are based on hybridization of precisely designed fluorescently labelled probes to their cellular targets. However, the conventional RNA FISH technique lacks enough sensitivity for low‐abundance lncRNA molecules. smRNA‐FISH was developed later to circumvent this problem, by making use of a pool of short probes designed to tile the entire length of lncRNA. smRNA‐FISH is very sensitive in detecting low‐abundance lncRNA transcripts, being quantitative and allowing absolute measurement of the transcripts 44, 45.

A common obstacle in revealing the subcellular localization of lncRNA molecules is the off‐target hybridization of probes to nuclear aggregations, generating blob‐like signals like that of true lncRNA signals. Cabili *et al*., made use of two pools of probes labelled with different fluorophores and co‐localizing the signals obtained using the two probe sets. If the signals co‐localize, then they are construed as true ones indicating the lncRNA localizations, otherwise at least some of the signals are considered to be due to off‐target hybridizations 46 (Table 1).

**Table 1:** Advantages and disadvantages of the techniques used to characterize lncRNAs

Approaches	Advantages	Disadvantages
Methods used to localize lncRNAs inside the cell		
1. sm‐RNA FISH	‐ Visual localization of the intracellular location of lncRNAs	‐ The possibility of false positive detection due to similarity of true nuclear lncRNA signals to possible off‐target nuclear signals
	‐ Detection of low abundance lncRNAs through probes that tile all of true nuclear lncRNA signals to possible off‐target over the transcript length	
	‐ Quantitative measurement of lncRNA transcripts	
2. CRISPR RNA Tracking determining	‐ Does not require manipulation endogenous RNA molecules	‐ Three components need to transfected into the cell
	‐ Does not affect the abundance or expression profile of endogenous RNAs	‐ Nuclear localization of GFP‐dCas9 may complicate the location of nuclear resident RNAs
Methods used to predict lncRNA structure		
1. SHAPE	‐ Capable of revealing single‐stranded and double‐stranded lncRNA motifs	‐ Inability to determine the range of base pairing interactions
		‐ Limited to *in vitro* experiments
2. PARS	‐ Capable of revealing the global structure of lncRNAs or the lncRNA structurome	‐ Limited to experiments done *in vitro* and cannot be done *in vivo*
	‐ Capable of revealing the global changes in lncRNA structure under different cellular conditions and presence of toxins or drugs	
3. dChIRP	‐ Increased signal to noise ratio due to recovery based on individual domains as compared to ChIRP	‐ Requires a prior knowledge of individual lncRNA domains
	‐ Revelation of lncRNA interactions with RNA, DNA and proteins in a single reaction unlike ChIRP and CLIP	
	‐ Characterization of individual lncRNA domains and their functions	
Methods utilized for determination of lncRNA function		
1. CRISPR/Cas9	‐ Less off‐target effect in comparison to conventional knockout methods	‐ Leading to introduction of indels by NHEJ which cannot ablate non‐coding genes
	‐ High recombination frequency	
	‐ Complete lncRNA perturbation in case of the dual gRNA system	
2. RNAi	‐ Low off‐target effect if ESI RNAs are used	‐ Stable silencing of target transcripts is not possible if esi‐ or si‐RNAs are used
	‐ Tighter intracellular level of silencing if ESI RNAs are used	‐ Inducible silencing is not possible if ESI or si‐RNAs are used
	‐ Cost effective	
	‐ More resistant to nucleases compared to ASO	
	‐ Inducible and stable silencing is possible if shRNAs are used	
3. CRISPRi	‐ Acts on DNA and therefore has a broader target range	‐ Needs transfection of several components into the cell
	‐ Can be better applied to noncoding transcripts	
	‐ Can be adapted for multiple silencing of several genes	
	‐ Active in both cytoplasm and nucleus	
4. ASO	‐ Capable of silencing nuclear lncRNAs	‐Being labile and sensitive to nucleases
	‐ The probe length can be fine‐tuned to achiev the optimal specificity unlike RNAi probes which have a constant length	‐Increased off‐target effect in case of modified ASOs
Methods Used for mapping lncRNA interactions		
1. CHART	‐ Reduced background signal owing to fewer number of probes used	‐ Formaldehyde used as the cross‐linking agent is not efficient
	‐ No prior knowledge of individual domains is required	‐ Probe synthesis is time consuming due to the RNase H assay required
2. ChIRP	‐ Reveling genome‐wide lncRNA DNA binding sites	‐ Increased noise‐to‐signal ratio in comparison to dChIRP
	‐ Prior knowledge of individual domains is required by using probes that tile all over the length of lncRNAs	‐ Unable to reveal the function of individual lncRNA domains
	‐ Short and easy‐to‐synthesize probes used decrease the off‐target effect	
3. RAP	‐ No prior knowledge of individual domains is required	‐ Long probes used which are difficult to synthesize and increase the off‐target effect
	‐ Capable of detecting residual lncRNA fragments resulting from shearing	
Mapping lncRNA‐protein interactions by protein pull‐down		
1. RIP	‐ No prior knowledge of the RNA partners is required in case of RIP‐seq	‐ Requires antibodies against known proteins
	‐ Genome‐wide mapping of RNA‐protein interactions	‐ Pull‐down of off‐targets due to nonspecific interactions
		‐ False negatives
2. CLIP	‐ Global screening of RNA‐protein interactions in HITS‐CLIP	‐ Pull‐down of nonspecific proteins
	‐ No prior knowledge of RNA is required	‐ Inefficiency of UV cross‐linking
	‐ Reduction of false negatives by using UV cross‐linking	‐ False positive detections that may occur due to nonspecific interactions during cross‐linking
	‐ Higher resolution in case of RNase digestion	
3. PAR CLIP	‐ Improvement of cross‐linking due to 4‐SU/6‐SG labeling	‐ Limited to cell culture systems
	‐ More accurate mapping of RNA‐protein interaction	‐ Pull‐down of nonspecific proteins
4. iCLIP	‐ Nucleotide resolution mapping of lncRNA‐protein interactions	‐ Nonspecific pull‐down of proteins
	‐ Detection of rare events owing to amplification	‐ Bias due to nonlinear PCR amplification
Next generation sequencing based methods for global investigation of lncRNAs		
1. LIGR‐seq:	‐ Maps RNA‐RNA interactions	‐ Hybrid ligation may be difficult between short RNA fragments
	‐ Does not need prior knowledge of interacting protein partner	
	‐ Implemented *in vivo*	
2. TRIBE‐seq	‐ Not dependent on antibodies for affinity purification	‐ Not capable of revealing exact protein‐RNA interface
	‐ Needs a small number of cells	‐ Results may be complicated due to endogenous ADAR activity
	‐ Reveals protein‐RNA interaction is a cell specific manner	
3. ICE‐seq	‐ Reveals global A‐to‐I editing events	‐ Unable to detect completely edited sites
	‐ The results are not confounded by Gs occurring due to sequencing errors	
	‐ Individual variations due to SNPs do not confound the results	
4. GRO‐seq	‐ Measurement of relative activity of sites undergoing transcription	‐ Introduction of artefacts during preparation of nuclei
	‐ Detection of divergent transcription	‐ New initiations that may occur ensuing abortive transcription
	‐ No prior knowledge of transcript initiation site is needed	‐ Limitation to cell culture for incorporation of labeled analogues
5. BRIC‐seq	‐ Measurement of the half‐life of lncRNAs	‐ Introduction of artefacts during preparation of nuclei
	‐ Revealing the identity of RNA molecules based on their half‐life	‐ New initiations that may occur ensuing abortive transcription
		‐ Limitation to cell culture for incorporation of labeled analogues

RNA FISH and immunofluorescence have been used in order to show the co‐localization of Urothelial carcinoma associated 1 (UCA1(and phosphorylated hnRNP1 in the perinuclear regions to confirm that the phosphorylated form of hnRNP1 is able to interact with UCA1 47. Dual RNA FISH and immunofluorescence using probes against MALAT1, U2‐snRNA, and antibodies against SR splicing factor, SRSF1, have been used to clarify the role of MALAT1 in RNA splicing. MALAT 1 was shown to be co‐localized to nuclear speckles with SR splicing factors 48.

### RNA Mimics of GFP and RNA visualization

RNA tracking methods have been developed that take advantage of green fluorescent protein fused to MS2 coat protein (MCP) and the MS2 binding sequence inserted into the RNA molecule to be analysed. The RNA molecule containing the MS2 binding site and the GFP‐MCP fusion protein are expressed inside the cell. The localization of RNA molecules is revealed through GFP‐MCP binding to the MS2 site 49. However, as the GFP‐MCP fusion protein contains nuclear localization signal sequences, it may affect the subcellular location of RNA molecules inside the cell. Furthermore, investigation of nuclear resident RNAs may become complicated due to accumulation of the GFP‐MCP inside the nucleus and intense nuclear fluorescence 41. These impediments have stimulated the development of newer and better techniques for locating RNA molecules.

The GFP fluorophore, 4‐hydroxybenzylidene imidazolinone (HBI) does not show fluorescence in the absence or denaturation of the GFP protein. RNA mimics of GFP are aptamer molecules that can bind HBI or other GFP like fluorophores and enable them to fluoresce 42. If inserted to 3′ end of RNA molecules including lncRNAs, the localization of target RNAs can be revealed upon addition of the fluorophore to the cell. One RNA mimic of eGFP referred to as Spinach binds DFHBI [(Z)‐4‐(3,5‐difluoro‐4‐hydroxybenzylidene)‐1,2‐dimethyl‐1H‐imidazol‐5(4H)‐one] was inserted to the 3′ end of 5S rRNA and successfully revealed its subcellular distribution 42. Therefore, the GFP RNA mimics can be applied to lncRNA research so as to enhance and simplify the attempt to map subcellular localization of these transcripts and how they might change in cancer disease.

### Clustered regularly interspaced short palindromic repeats (CRISPR) RNA Tracking

The CRISPR/Cas9 system is considered as a type of adaptive immunity in bacteria and archaea 50. The Cas9 protein is the effector nuclease in type II CRISPR systems, which targets and cleaves invading phage DNA in close proximity to a sequence called protospacer adjacent motif (PAM) 51. The Cas9 protein from type II CISPR system was believed to recognize only DNA but not RNA sequences 52. However, a study performed by the Doudna laboratory in 2014 revealed the ability of Cas9 protein to target and cleave RNA sequences, therefore, expanding the biotechnological utility of this protein from DNA to RNA 52. The Cas9 protein was shown to bind RNA in the presence of a guide RNA. However, no RNA cleavage activity was observed until addition of a short oligonucleotide sequence (called PAMmer) with an NGG PAM sequence at its 5′ end. Furthermore, the binding affinity was shown to increase fivefold in the presence of the PAMmer 52.

**Figure 2:**
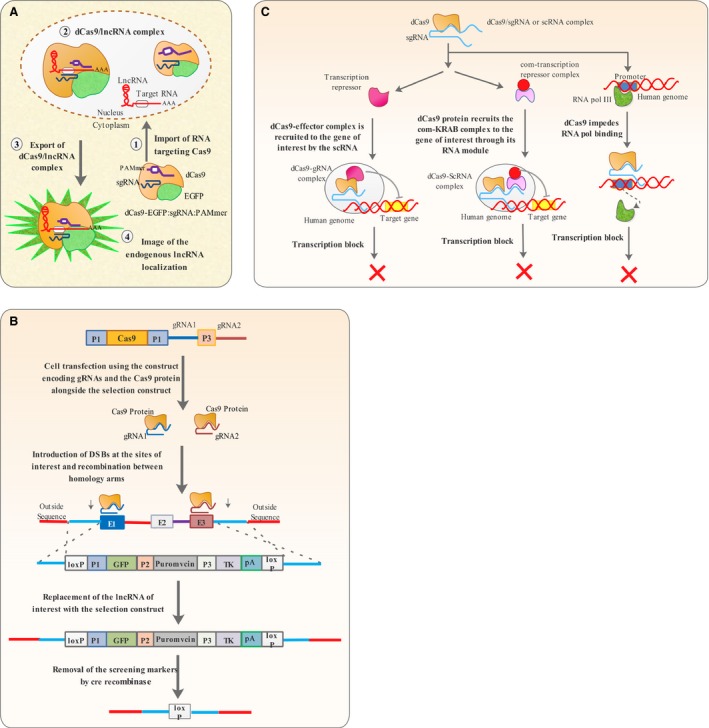
CRISPR‐based technologies. (**A**) CRISPR RNA tracking utilizes a catalytically dead Cas9 protein (dCas9) in the presence of gRNA and PAMmer oligonucleotide to visualize the subcellular location of RNA molecules. (**B**) The all‐in‐one system CRISPR knock‐out strategy uses a two gRNAs that direct Cas9 nuclease activity to both ends of a given lncRNA, which is to be replaced by a construct comprising GFP, neomycin and tk, flanked by loxP sites. After screening using neomycin and GFP the DNA construct in excised out using cre and leaves a loxP site in the lncRNA locus, something confirmed by negative selection using tk. **C.** CRISPRi suppresses the process of transcription by directing or recruiting chromatin modifying proteins to the gene of interest or by directly blocking RNA polymerase binding.

The RNA‐binding capability of Cas9 protein has spurred the development of technologies for tracking the subcellular localization of RNA molecules 53. A nuclease null Cas9 protein fused to EGFP (dCas9‐EGFP) was shown to successfully recognize ACTB, CCNA2 and TFRC mRNAs only in the presence of gRNA 53. However, dCas9 binding affinity to RNA was shown to be augmented in the presence of the PAMmer sequence 52, 53. In the presence of gRNA and PAMmer components recognizing the 3′ UTR of mRNA molecules, the nuclear localized dCas9‐EGFP protein was found to be co‐exported to the cytoplasm and reveal the subcellular localization of these RNA molecules, with comparable success to experiments performed with FISH on the same RNA molecules 53 (Fig. 2A). Furthermore, the CRSPR‐based RNA tracking technology was also shown to be capable of locating the ACTB mRNA in stress granules, as both the dCas9‐EGFP and Ras GTPase‐activating protein‐binding protein 1 were found to be merged in such granules 53.

Despite being tested on few mRNA molecules, the CRSPR‐based RNA tracking technology has the potential to be utilized in studies aimed at revealing the subcellular localization of RNAs including lncRNAs.

Unlike RNA detection technologies based on RNA–protein interactions, the CRSPR‐based RNA tracking technique does not require laborious genetic engineering procedures, as target recognition is based on RNA–RNA interactions 54. In addition, unlike RNA tracking methods based on MCP protein (described in 1.2.), this system does not require modification of the target RNA molecules and has no significant effect on localization, abundance or expression profile of its target RNAs 54 (Table 1). Therefore, CRISPR RNA tracking can be a complement to current methods of RNA tracking, providing new opportunities for better tracking and visualization of lncRNAs localization and its likely aberrations involved in cancer disease.

## Methods used to predict lncRNA structure

LncRNAs are capable of folding on themselves and therefore taking a wide variety of secondary and tertiary structures, enabling them to perform their functions 55. Therefore, an understanding of structure is an indispensable part of lncRNA research 55. There are several techniques available to allow the structural and conformational investigation of lncRNAs.

### Selective 2′‐hydroxyl analysed by primer extension (SHAPE)

**Figure 3:**
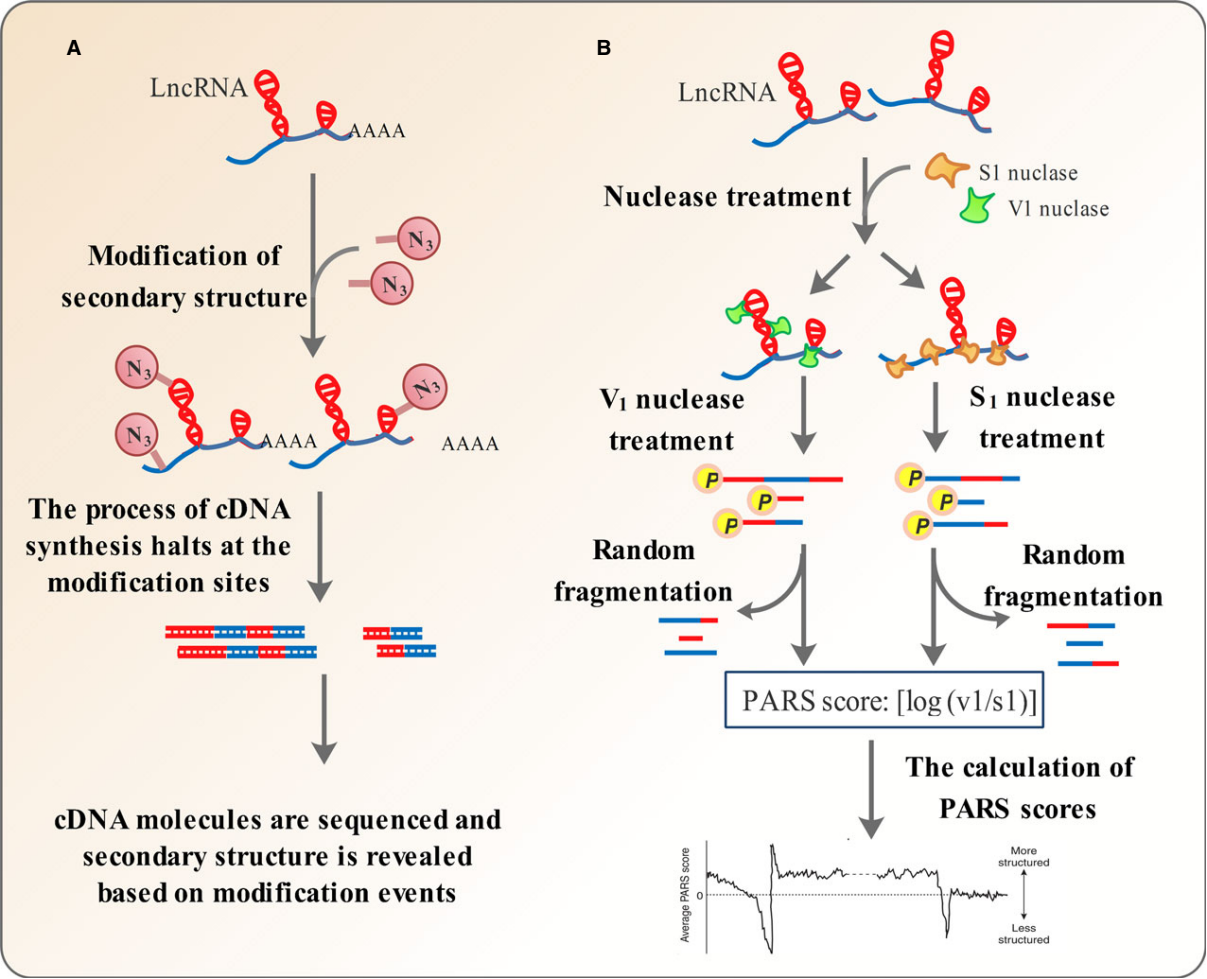
Methods used to predict lncRNA structure. (**A**) In SHAPE, flexible regions of RNA sequence are modified using chemicals that react with 2′ hydroxyl groups in single‐stranded regions (Acylation). Then, cDNA synthesis is performed but halts at positions, which are chemically modified. Finally, reverse transcription stop positions are determined using deep sequencing to reveal RNA secondary structure. (**B**) In PARS, the lncRNA molecules are refolded *in vitro* and treated by V1 and S1 ribonucleases specific for double‐ or single‐stranded RNA, respectively, in separate reactions. The resulting digested RNA molecules have phosphates at their 5′ ends. The fragments are then ligated to adaptors at their 5′ ends, subjected to random fragmentation and deep parallel sequencing. In each case, the RNA parts digested by the nucleases are the predominant parts, which could be ligated to adaptors and therefore sequenced. Finally, a PARS score is obtained as the ratio of double‐ *versus* single‐strandedness. A higher PARS score indicates a greater probability of nucleotides to be in double‐stranded regions, and vice versa.

SHAPE is among the key techniques commonly used to unravel the secondary structure of lncRNAs 56. The technique makes use of the chemical N‐methylisatoic anhydride (NMIA) that acylates single‐stranded RNA and depends on the fact that 2′ hydroxyl groups in single‐stranded or flexible regions of RNA are highly reactive 56. The chemical modification of single‐stranded RNA hinders the reverse transcription process in flexible regions 56. Therefore, the parts of RNA which could undergo reverse transcription are sequenced and considered as to be involved in secondary structures, while other parts are interpreted to be single‐stranded 56 (Fig. 3A).

Despite being a powerful technique in unravelling the single and double‐stranded regions of lncRNA molecules, SHAPE is not able to determine the range of such base paring interactions 57. The shotgun secondary structure (3S) determination technique has been developed to overcome this problem by fragmenting RNA molecules before SHAPE is done 57. Finally, the data from such fragmented transcripts are compared to whole transcript SHAPE data to provide insights into the low range and high range base pairings within the secondary structure 57. Probably due to its short half‐life in water and cross‐reactivity with other nucleophiles abundant in the cell, the conventional electrophile used in SHAPE is not applicable to structural analysis of RNA molecules *in vivo* 58 (Table 1). Screening of a variety of electrophilic compounds led to the identification of 2‐methyl‐3‐furoic acid imidazolide (FAI) and 2‐methylnicotinic acid imidazolide (NAI), which could meet all the parameters needed for extending the applicability of SHAPE from *in vitro* to *in vivo in* icSHAPE(*in vivo* click SHAPE) 58.

An array of chemical probing techniques including SHAPE has been employed to characterize HOTAIR in structural terms 59. The resulting structural map revealed that HOTAIR is made of four modules, with two of them serving as the protein‐binding domains. This HOTAIR map also indicates several structural motifs conserved in the course of evolution 59. The capability of SHAPE to reveal the single‐stranded lncRNA parts can be served to clarify lncRNA–mRNA interactions 60. SHAPE was used to show that the lncRNA, TINCR have single‐stranded regions that are complementary to sequence motifs on its target mRNA, thereby indicating a case in which mRNAs can be regulated by an lncRNA through base pairing 60.

### Parallel analysis of RNA structure (PARS)

Structural studies of ncRNAs in a single‐molecule manner have been carried out successfully 61. Two methods with similar mechanisms named PARS 62 and Frag‐seq 63 were introduced recently, for global mapping of coding and non‐coding RNA transcripts. The PARS technique utilizes RNase proteins specific for single‐ or double‐stranded motifs in RNA molecules, leaving behind a 5′ phosphate. RNA molecules are refolded *in vitro*, subjected to RNase treatment, ligated to adaptors and sequenced. Finally, a PARS score is obtained as the log ratio of in a double‐ or single‐stranded conformation. The log ratio between the number of sequence reads obtained for each nucleotide in the V1 (cleaving double‐stranded RNA) and S1 (cleaving single‐stranded RNA) experiments. A nucleotide with a higher log ratio, or PARS score, thus indicates a higher probability of being in a double‐stranded conformation (Fig. 3B). PARS is capable of expanding the boundaries of RNA structural analysis to the realm of high‐throughput 62. However, like many other techniques for analysing RNA structural features, PARS is limited to studies performed *in vitro* and cannot be employed *in vivo* 64 (Table 1).

PARS has been successfully applied to coding and non‐coding RNA molecules, and structural evaluation of two domains from the HOTAIR lncRNA was successfully carried out using this technique 62. The systematic probing of RNA structure using PARS under different chemical or physical conditions or in the absence or presence of other molecules can provide valuable insights into RNA structural changes that can affect cellular behaviour 64 Therefore, these techniques have the potential to be used in evaluating the likely changes in lncRNA structurome that may occur in cancer disease and screening of compounds that may restore such changes to the normal state.

### Domain‐specific chromatin isolation by RNA purification (dChIRP)

As pivotal regulators of gene expression, lncRNAs have been poorly characterized regarding their domain architecture. Domain‐specific chromatin isolation by RNA purification (dChIRP) is a modification of ChIRP (described in 4.1.1) that allows simultaneous mapping of RNA–RNA, RNA–DNA and RNA–protein interactions at the level of individual domains 65.

In this technique, the cells are subjected to cross‐linking agents to preserve protein–nucleic acid interactions and then sonicated to shear the nucleic acids. Specific biotinylated oligonucleotides are used to tile individual domains. Streptavidin magnetic beads are then added to capture specific interactions and non‐specific interactions are washed away. The material recovered from dChIRP is finally fractioned into protein, DNA and RNA parts for extraction and analysis. The parts can be subjected to real‐time PCR using primers for the lncRNA domain of interest and other RNAs suspected to be in its interaction. The protein content can be analysed by Western blotting or mass spectrometry to reveal any protein interacting with different domains. Recovered DNA can be also sequenced, revealing any binding sites on genome that may become occupied by a given lncRNA domain 66.

Unlike other techniques used to map lncRNA interactome, dChIRP is capable of revealing lncRNA multivalent interactions with other RNA, DNA and protein molecules in a single reaction 65 (Table 1).

Using dChIRP, Rox lncRNA was indicated to have interaction with MSL proteins through its D1, D2 and D3 domains. The Rox binding sites on X‐chromosome (dlg1, suv4‐20 and u2af50) were also clearly determined. dChIRP has the potential to be applied to other lncRNAs with important implications in cancer, on the way to revealing their interactions in a domain by domain manner 66. Furthermore, dChIRP performed on MDA‐MB‐231 breast cancer cells was indicated to be capable retrieving HOTAIR domain‐specific RNAs, proving the generality of this technique 67. These techniques can provide valuable insights into which lncRNA parts are the culprits in disrupting its overall function and narrow down the therapeutic strategies to focus on specific modules rather than the whole molecule.

## Methods utilized for determination of lncRNA function

The traditional approach for gene discovery called forward genetics relies on finding aberrant phenotypes in a model organism and then unravelling the genes behind such phenotypes. In reverse genetics, however, a given gene of interest is perturbed and the resulting phenotypic change is observed, helping to find the gene function. Reverse genetics are particularly important in delineating the important roles of lncRNAs in cancer, as gene ablations can clearly depict their contributions to cancer phenotype or transformation of normal cells. Here, we look at the techniques utilized in lncRNA reverse genetics 68.

### Knockout techniques

#### Clustered regularly interspaced short palindromic repeats (CRISPR) Cas technology

Several technologies for genome editing based on programable proteins including Zinc‐finger nucleases (ZFNs),transcription activator like effector nucleases (TALENs) and Cas9 have emerged over the past 10 years 69. These proteins can be engineered to recognize specific DNA sequences and introduce double‐stranded breaks. TALEN and ZFN specificities are based on protein–nucleic acid interaction 69. The TALE domain of TALEN can be engineered to recognize different DNA sequences and fused to FokI restriction nuclease. The TALE domain then guides FokI to the site of interest where it induces a double‐stranded break 69. The Cas9 specificity is based on RNA–DNA interaction, and the protein itself has intrinsic nuclease activity 51. Genome editing based on ZFN and TALEN is more cumbersome compared to Cas9 as it requires arduous genetic engineering 69. Unlike coding genes, it is much more difficult to knock‐down non‐coding sequences, including lncRNAs 70. Gene knockout based on programmable nucleases could not be applied to non‐coding sequences efficiently, because lncRNAs can yet remain functional even after insertions or deletions 71. Ho and his colleagues developed an all‐in‐one system, comprising two gRNAs and the Cas9 protein to overcome this hurdle. The gRNAs are capable of directing the Cas9 endonuclease to the boundaries of lncRNA encoding sequence; therefore, the whole sequence will be replaced by a synthetic construct by homologous recombination 70 (Fig. 2B). Non‐homologous end joining (NHEJ) is also an impediment towards perturbing non‐coding sequences due to its competition with homologous recombination, therefore, leading to introduction of indels which are insufficient to completely disturb the function of lncRNAs. Coupling the all‐in‐one system strategy with siRNAs against Ku70, Lig4 and XRCC4 (proteins involved in NHEJ) can increase HR to NHEJ ratio and therefore lead to complete knock‐down of lncRNA of interest 70.

Making use of two gRNAs, the dual CRISPR system is suited for perturbing lncRNAs in contrast to conventional methods, which may lead to insertions or deletions, not enough to eliminate the function of lncRNAs 70, 72 (Table 1).

The applicability of the all‐in‐one system strategy in perturbing lncRNAs was illustrated by ablating urothelial carcinoma‐associated 1 (UCA1), lncRNA‐21A and AK023948 in various human cell lines 70. In another study, PRNCR1 and PCGEM1 were perturbed and indicated to be involved in inhibiting PYGO2 function in AR‐dependent promoter/enhancers in castration‐resistant prostate cancer 73. However, PRNCR1 and PCGEM1 were then shown not to be involved in castration‐resistant prostate cancer 74. A dual knockout system was also used to eliminate the promoter region of MALAT1 so as to block its transcription in a number of human cell lines 75. Therefore, genome editing technologies including CRISPR/Cas9 are key tools in reverse genetics of lncRNAs and herald a new era in which many lncRNAs can be deleted systematically so as to provide a coherent picture of their role in cancer 76.

### Knock‐down techniques

#### RNA interference (RNAi)

While the knockout techniques eliminate gene expression by deleting the genes, RNAi suppresses expression by sequestering and then cleaving RNA molecules 77, 78. Several knock‐down strategies are in common use, utilizing either synthetic or enzymatically prepared RNAi libraries 79. Endoribonuclease‐prepared siRNAs are made by treatment of dsRNAs with RNase III or Dicer 79. The resulting RNAs, called esi‐RNAs, are delivered directly into the cytoplasm. Esi‐RNAs show less off‐target effect and trigger a tighter suppression of target RNAs compared to conventional methods 79. The silencing response using esi‐RNAs is transient 79, 80, 81. Conventional siRNAs are prepared by chemical synthesis and delivered into cytoplasm directly 79. Chemically synthesized siRNAs also bring about a tight silencing response, despite showing a more off‐target effect compared to esi‐RNAs. The response triggered by siRNAs is transient 79. shRNAs are another class of RNAs used in RNAi. They are not delivered directly to the cytoplasm but are expressed inside the cell. The silencing response triggered by shRNAs may be transient or stable, and the off‐target effect is greater compared to esi‐RNAs 79 (Table 1).

Any RNAi experiment has to be accompanied by standard controls 82. Oligonucleotides recognizing no targets inside the cell have to be used alongside the RNAi experiment so as to ensure that the resulting phenotypic change is not due to non‐specific recognition of other targets 82. If the control siRNA indicates no significant change in cell viability or phenotype compared to control, the resulting changes in cells treated with test siRNA are specific 82.

Involved in aggressive prostate cancer, second chromosome locus associated with prostate‐1 (SChLAP1), is an lncRNA that inhibits the tumour‐suppressive activity of the SWI/SNF complex. The role of SChLAP1 in contributing to aggressiveness of prostate cancer was assayed by performing siRNA‐mediated knock‐down experiments. Remarkably, the SChLAP1 knock‐down dramatically decreased cell proliferation and invasiveness.^35^. siRNAs against UCA1 and hnRNP1 were used in different experiments to show that UCA1 knock‐down is associated with increased p27 expression, while hnRNP1 depletion reduces p27 expression. These observations confirmed the oncogenic role of UCA1 in breast cancer through suppressing p27 expression.^47^.

RNAi has been successfully applied to many loss‐of‐function studies involving lncRNAs 71. However, when a nuclear lncRNA is to be targeted, RNAi is not efficient furthermore, although the RNAi machinery has been shown to be active in the nucleus 71, 83.

#### CRISPR Interference (CRISPRi)

The versatility of CRISPR/Cas9 has enabled repurposing of its utility from genome editing to genome regulation 84. Recently, a modified CRISPR system called CRISPRi has been proven to be effective in down‐regulating gene expression by blocking transcription 84. A minimal CRISPRi machinery consists of a catalytically dead Cas9 protein lacking endonucleolytic activity (dCas9) and a gRNA recognizing the target gene 84. gRNAs targeting the non‐template DNA strand in promoter or ‐35 regions are more effective in gene down‐regulation than those targeting template strand sequences or regions 100 bp upstream of the promoter 84 (Fig. 2C). Despite being quite efficient in repressing gene expression in bacteria, CRISPRi based on direct blocking of RNA polymerase was not as efficient when applied to eukaryotic cells 84. This problem led researchers to design chimeric dCas9 proteins fused to chromatin modifying effectors, including KRAB (Kruppel‐associated box) so as to bring about epigenetic silencing of gene expression 85. This new CRISPRi platform was indicated to successfully repress the expression of *GFP* in HEK293 cells and endogenous genes including *CD71, CXCR4* in HeLa cells 85. CRISPRi through epigenetic silencing has been further refined by making use of protein‐binding RNA modules that bind and recruit the effector protein instead of direct fusion of effector to dCas9 84. Well‐characterized protein‐binding RNA modules including MS2, PP7, and com recognized by MCP, PCP, and com RNA‐binding proteins can be fused to the 5′ end of gRNA to make scaffold RNA (scRNA) 84. Genes can be down‐regulated by making use of an scRNA that can recruit any given RNA‐binding protein fused to any chromatin modifier, com‐KRAB fusion, for instance, to the site of interest. 84 In addition, simultaneous silencing of several genes can be brought about by utilizing several scRNAs consisting of com modules and gRNAs targeting different loci, therefore, recruiting com‐KRAB fusion to target sites and repressing their expression 84.

Acting at the DNA level, CRISPRi can be a useful tool to target non‐coding transcripts including lncRNAs as well as microRNAs and products of RNA polymerase III 85. The CRISPRi system has a greater target range compared to RNAi and does not compete with endogenous RNA machinery and therefore can complement the current tools at our disposal 85 Knock‐down studies are valuable resources in studying the role of different cellular components including lncRNAs and the CRISPRi holds a great promise for knock‐down experiments aimed at revealing the role lncRNAs behind different cancers due to its capability to repress transcription at the DNA level and to repress multiple genes simultaneously 85 (Table 1).

#### Combined knock‐down and localization analysis of non‐coding RNAs (c‐KLAN)

RNA‐FISH is utilized to localize RNA transcripts inside the cell 43, whereas RNA interference is utilized in functional studies 78. Despite being both highly useful and revealing, these techniques bear disadvantages including off‐target silencing 79. Endoribonuclease‐prepared siRNAs have been developed to remove the drawbacks of off‐target detection and difficulty of production associated with conventional RNAi 79. esi‐RNA production protocol can be also employed for production of labelled probes against lncRNA targets from cDNA pools 79. c‐KLAN combines RNA interference and localization techniques to shed light on both location and function of lncRNAs, paving the way to their robust characterization. cDNA is first amplified using PCR. Then, *in vitro* transcription in the presence of labelled UTPs can be performed for the generation of RNA FISH probes. *In vitro* transcription followed by RNase III digestion can be implemented for the production of esi‐RNAs. c‐KLAN combines the power of RNA FISH and RNAi for characterizing lncRNAs through loss‐of‐function studies and subcellular visualization 86.

c‐KLAN has been efficiently utilized to reveal the lncRNAs involved in pluripotency 86. Pluripotency‐associated non‐coding transcript 1 has been successfully found to be the major lncRNA involved in this state, using Oct4‐GiP cells that express GFP under Oct4 promoter 86.

#### Antisense oligonucleotides (ASOs)

Despite being highly effective devices in loss‐of‐function studies, siRNA and shRNAs cannot be used effectively in depleting nuclear lncRNAs, although the RNAi machinery also exists in the nucleus 71, 87, 88.

As effective tools for silencing nuclear lncRNAs, ASOs are modified or unmodified single‐stranded deoxyribonucleotides, with the ability to hybridize to their targets among a population of transcripts present in the cell. Knock‐down experiments using ASOs are based on the ability of RNase H to degrade the RNA part of a DNA–RNA duplex 89. Single‐stranded antisense oligonucleotides are similar to siRNAs in that they interact with their targets through Watson–Crick base pairing 89. However, unlike siRNAs, ASOs are labile inside the cell due to sensitivity of single‐stranded DNA molecules to nucleases, a problem that led to the development of gapmers 89. Gapmer oligonucleotides are modified ASOs harbouring 2–5 chemically modified nucleotides (e.g. locked nucleic acid (LNA) or **2**′‐O‐methoxyethylribose (2′‐MOE)) on their termini, intervened by a central ‘gap’ of unmodified DNA, 8‐10 bases in size 89. The modified nucleotides confer nuclease resistance to gapmers, while the central DNA sequence allows them to bind their targets. However, modified antisense oligonucleotides cannot bind their targets efficiently, a problem that forces the use of a higher concentration of ASOs, leading to more off‐target effects 89 (Table 1).

Several groups have used ASOs successfully in loss‐of‐function studies of nuclear lncRNAs. For instance, ASOs were utilized in an attempt to knock‐down the nuclear‐retained MALAT1, systemically. Two gapmers against different parts of MALAT1 were used, and systemic knock‐downs using ASOs were shown to recapitulate the same results observed in knockout studies. Besides, the same reduction in metastasis happening upon genetic loss of *MALAT1* was also observed in systematic MALAT1 depletion using ASOs 90.

## Methods used for mapping lncRNA interactions

Cell function is governed by a complex network of interactions involving different cellular components 91. Important among such components are lncRNAs, playing a wide variety of roles through their interaction with other RNA and protein molecules 91. Any aberration in such interactions can have dire consequences including cancer 91. Therefore, mapping lncRNA interaction networks is an indispensable part of any research in cancer biology. In this section, we summarize the techniques employed commonly in mapping lncRNA interactions.

### Mapping lncRNA–DNA interactions

#### ChIRP, CHART and RAP as RNA‐centric methods to map lncRNA genomic binding sites

**Figure 4:**
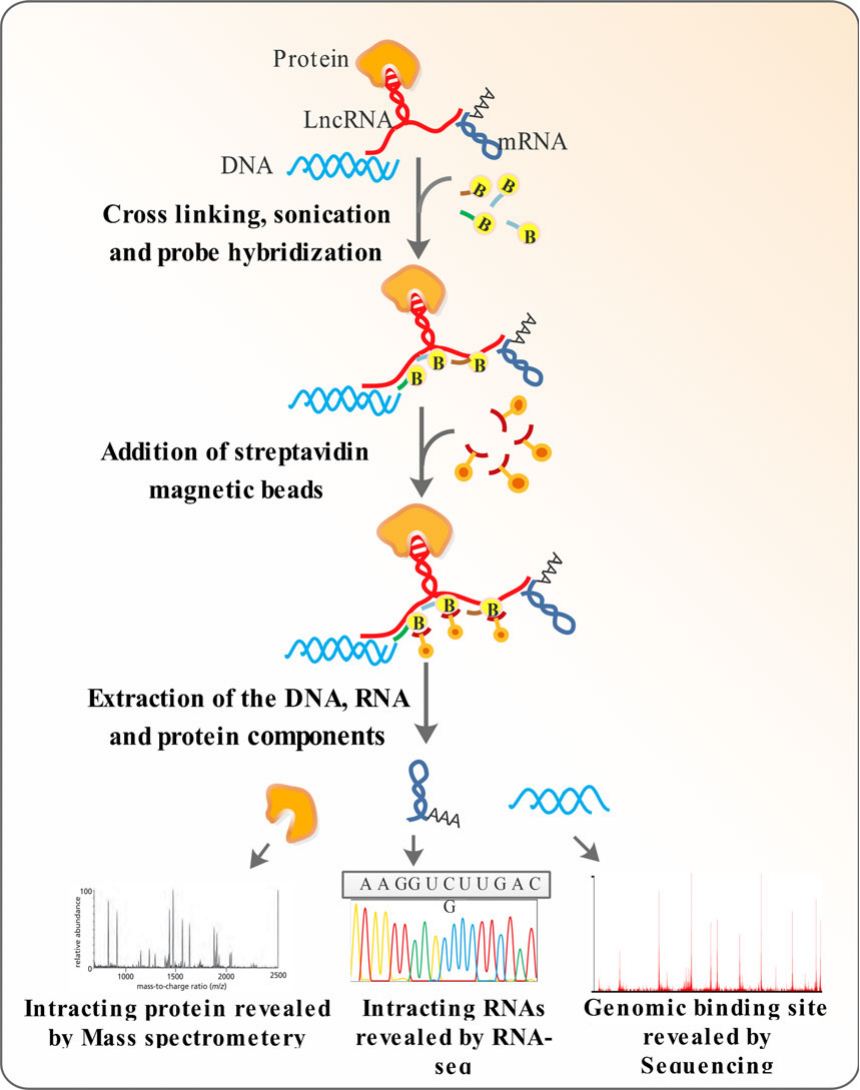
Mapping lncRNA–DNA interactions. In ChIRP, the lncRNA molecules are cross‐linked to their interacting partners including DNA, proteins and other RNA molecules. Biotinylated probes of about 20 bp in size are the used to tile the entire length of the lncRNA. Streptavidin magnetic beads are added to pull‐down the lncRNA molecules and their interacting components by binding to the biotinylated probes. The precipitated material is finally partitioned to DNA, RNA and protein fractions. ChIRP can be coupled to RNA‐seq (ChIRP‐seq) or mass spectrometry (ChIRP‐MS) for analysing the nucleic acid or protein fractions, respectively. (RAP and CHART are similar to ChIRP in their overall procedure except for the probes of different size and different cross‐linking agents used in these techniques).

Conventional methods of chromatin precipitation commonly make use of antibodies against proteins associated with DNA 92. ChIRP (chromatin isolation by RNA purification) 93, 94, CHART (capture hybridization analysis of RNA targets) 95 and RAP (RNA antisense purification) 93 are RNA‐centric techniques that benefit from probes against RNA molecules to precipitate chromatin to reveal the lncRNA genomic binding sites 96, 97. The three techniques are similar regarding the overall procedure 96. After cross‐linking the cellular components, the lncRNAs are captured with biotinylated antisense probes. The RNA molecules are recovered using streptavidin‐linked magnetic beads, and the precipitate is fractioned into protein, RNA and DNA parts. The RNA and DNA fractions are finally analysed using RT‐PCR and high‐throughput sequencing, revealing the genome‐wide binding sites of the lncRNA of interest (Fig. 4) 96, 97. The proteins associated to a given lncRNA molecule can be also identified by subjecting the protein fraction recovered to mass spectrometry in ChIRP‐MS and RAP‐MS 96. However, the techniques have differences with respect to cross‐linking mode, the size and design of probes used 96. ChIRP and RAP make use of probes that cover all the length of lncRNA sequence, therefore, targeting all potential sites unmasked for hybridization 96. Probes used in ChIRP are shorter (about 20 bp in size) and therefore easier to synthesize and potentially discriminate against off‐targets, whereas longer probes costly to synthesize (about 120 bp in size) are used in RAP 96. CHART is similar to ChIRP but makes use of an RNase H assay to find experimentally the best target site for designing probes (18–28 bp in size) 93. The cross‐linking mode is another point of difference between the three methods, with glutaraldehyde used in ChIRP, formaldehyde employed in CHART and a combination of them utilized in RAP 93, 96. Longer cross‐linking agents such as glutaraldehyde have been proven to be more efficient than the smaller ones such as formaldehyde 93 (Table 1).

ChIRP, which can be considered as an analogue of ChIP, has been used by different research groups hitherto to map the genomic binding sites of different lncRNAs 93, 94, 96. ChIRP, RAP and CHART have been employed in a wide variety of studies on lncRNAs involved in cancer (Supplementary Table 1). For instance, Chu and coworkers were the first group that used ChIRP to reveal the genome‐wide DNA binding sites of HOTAIR lncRNA in MB‐231 breast cancer cells. They found 832 HOTAIR occupancy sites throughout the genome, located on multiple chromosomes and enriched in regions notably annotated as promoters and enhancers. 98.

### Mapping lncRNA–protein interactions by protein pull‐down

#### RNA immunoprecipitation (RIP)

RNA immunoprecipitation (RIP) is a technique which can be employed to unravel all lncRNAs that interact with specific proteins of interest 29, 99. The technique can be implemented with or without cross‐linking 100. UV irradiation can be also used for detection of direct RNA–protein interactions by RIP 100. The RNA–protein complexes are co‐immunoprecipitated using antibodies against the protein component 100. The RNA component is then recovered and subjected to sequencing, microarray or RT‐PCR 100. RIP combined with microarray or sequencing is referred to as RIP‐chip or RIP‐seq, respectively 48, 98, 100.

Like other protein‐centric pull‐down assays, RIP is dependent on antibodies previously produced against the suspected counterpart proteins 100 (Table 1).

In a study of the role of the lncRNA, UCA1, in breast cancer promotion, RIP was employed to indicate that UCA1 interacts with hnRNP1 and suppresses the p27 protein level by competitive inhibition 47. Then, they performed RIP after UCA1 expression and showed a reduction in p27 mRNA pull‐down level using antibodies against hnRNP1 47.

To investigate the role of MALAT 1 in splicing of pre‐mRNAs, antibodies against SRSF1 splicing factor, and primers specific to MALAT1 were used to reveal a specific interaction between them, using the RIP technique 48. In addition, RIP has been also utilized to show SChLAP1 but not other cytoplasmic lncRNAs are immunoprecipitated by SWI/SNF complex 35.

#### Cross‐linking and immunoprecipitation (CLIP)

**Figure 5:**
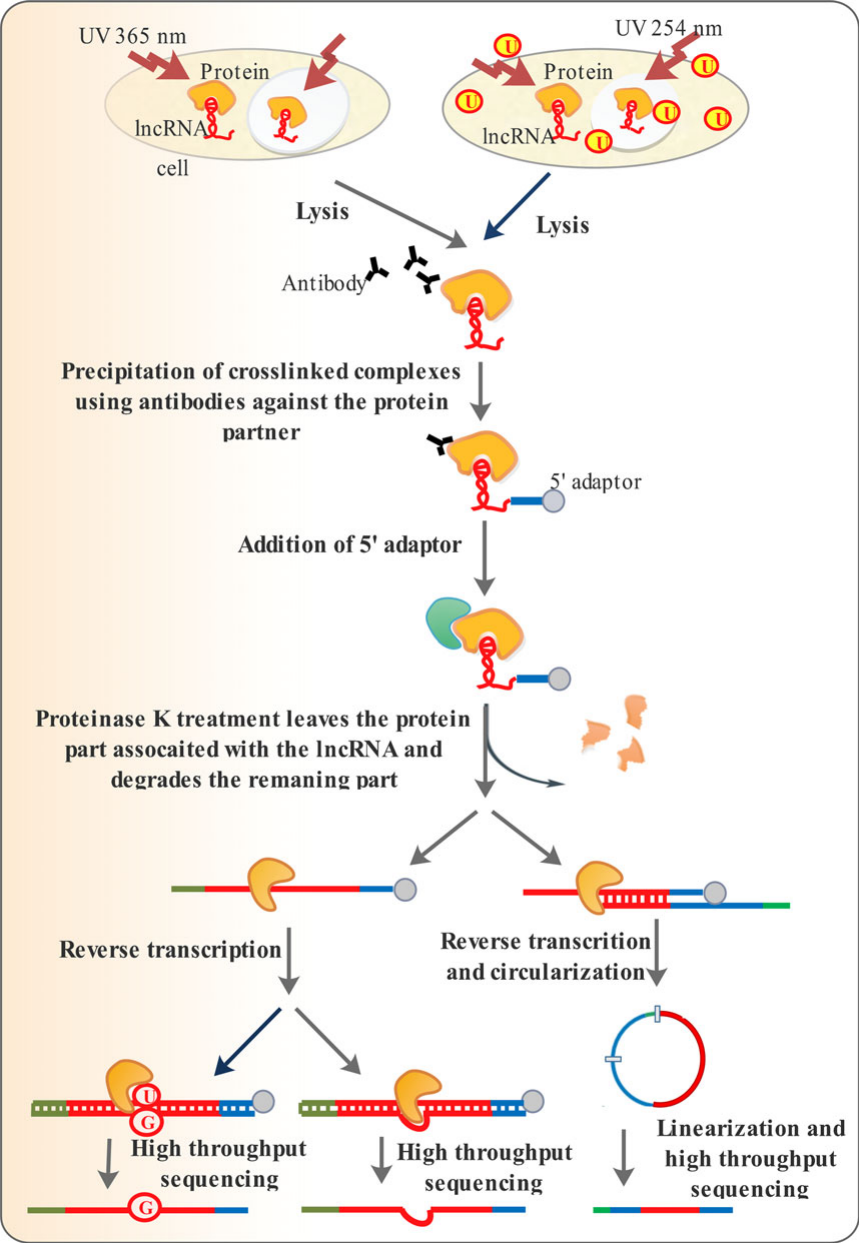
Mapping lncRNA–protein interactions by protein pull‐down. High‐throughput sequencing of RNA isolated by ultraviolet (UV) cross‐linking and immunoprecipitation (HITS‐CLIP), iCLIP and PAR‐CLIP all make use of specific antibodies for affinity purification a protein of interest. To co‐purify the associated RNA counterparts HITS‐CLIP and iCLIP use irradiation at 254 nm, while PAR‐CLIP employs irradiation at 365 nm using a photolabile 4‐thiouridine. Treatment with proteinase K degrades all protein parts but leaves a tiny RNA‐linked region. Following reverse transcription, the protein‐linked RNA site leads to insertions in HITS‐CLIP, results in truncation of cDNAs in iCLIP and causes base transition in PAR‐CLIP because of 4‐thiouridine analogue used to enhance cross‐linking. Deep sequencing of the cDNAs following PCR allows identification of the protein‐RNA interaction sites with single‐base resolution.

Further refinement of RIP has led to cross‐linking and immunoprecipitation (CLIP) technique, which is capable of revealing a more accurate picture of lncRNA–protein interfaces with single nucleotide resolution 101. In this technique, the lncRNA and protein components are cross‐linked; the complex is precipitated, and the RNA counterpart is identified 102. There are several versions of the CLIP technique, including photoactivatable‐ribonucleoside‐enhanced cross‐linking and immunoprecipitation CLIP (PAR‐CLIP) 103 and individual‐nucleotide‐resolution CLIP (iCLIP) 101. Both PAR‐CLIP and iCLIP are based on the propensity of some cross‐linked amino acids to remain associated with the RNA component after cross‐linking and include a reverse transcription step in which the precipitated RNA is converted to cDNA 102 (Fig. 5). In PAR‐CLIP, the RNA–protein interaction sites are revealed based on nucleotide misincorporation at the sites where such interactions occur 102. However, detection of nucleotide misincorporation sites requires the process of reverse transcription to go into completion, something that does not occur efficiently as the reverse transcriptase halts before the misincorporation point 104. To overcome this problem, size‐selected cDNAs are circularized and sequenced in iCLIP, and the truncation points in cDNA synthesis reveal the RNA–DNA interfaces 101.

In contrast to other CLIP variants, which makes use of cross‐linking with 254 nm UV light, PAR‐CLIP takes advantage of 4‐thiouridine to facilitate the cross‐linking process, allowing it to occur at a higher UV wavelength (365 nm) 102 (Fig. 5).

irCLIP and eCLIP are newly introduced versions of CLIP techniques developed to increase reverse transcription efficiency and decrease RNA loss and sequencing backgrounds 105. irCLIP makes use of a thermostable reverse transcriptase that functions at 60 °C to avoid reverse transcription stoppage due to RNA secondary structures 105. The utilization of Circligase II in irCLIP has allowed the reverse transcription and circularization steps to be combined as a single step, enabling the method to be performed in less than half the time needed in iCLIP 105. In eCLIP, a 5′ adaptor has been utilized to avoid RNA loss and a 3′ adaptor followed by a barcoded used to distinguish between unique reads and PCR duplicates 106.

The advantages and disadvantages of each of the CLIP techniques have been reviewed in (Table 1).

In a genome‐wide analysis of lncRNA binding sites on different RBPs, CLIP‐seq was employed to show the interaction of Ezh, a PRC2 complex subunit, with 35 lncRNAs including Tsix, Meg3, Rian and Pvt1 107.

Using PAR‐CLIP, TUG1, DLEU2 and GAS5 lncRNAs were also shown to bind at least two of the three RNA‐binding proteins HuR, Ago2 and MOV10 at identical binding sites 107.

Moreover, CLIP has been employed to show air interactions with H3K9 histone methyl‐transferase G9a 108.

## Next‐generation sequencing‐based methods for global investigation of lncRNAs

Over the past 20 years important efforts have been made allowing the research to be directed at transcriptome‐wide analysis of RNAs and changes in their global patterns of expression 40. Next‐generation sequencing technologies lie at the heart of such efforts as they enable bulk RNA analysis through massive cDNA sequencing 109. Transcriptomics strives to catalogue all RNA species inside the cells, delineate the splicing and other post‐transcriptional modifications or editing events the transcripts may undergo and to provide a global picture of the changes in transcriptome during development or disease conditions 109. Here, we will cover some of the technologies based on RNA‐seq developed to tackle such transcriptome‐wide questions.

### LIGation of interacting RNA followed by high‐throughput sequencing (LIGR‐seq)

**Figure 6:**
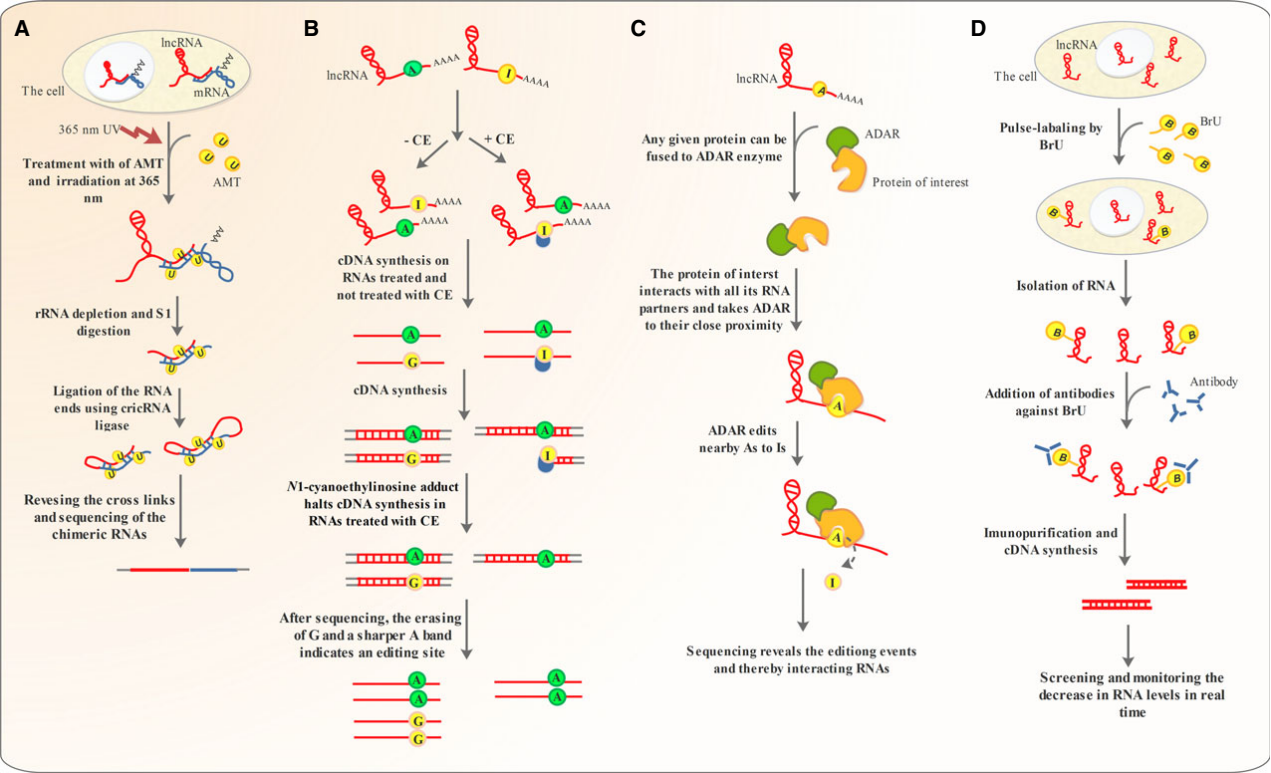
NGS‐based technologies used for global investigation of lncRNAs**.** (**A**) LIGR‐seq makes use of an intercalating agent and UV irradiation to join interacting RNAs. The RNA ends are partially digested and ligated using cricRNA ligase. The cross‐linking is reversed, and the chimeric RNAs are sequenced. (**B**) ICE‐seq exploits cyanoethylation with Acrylonitrile to convert Inosine to *N*1‐cyanoethylinosine, which halts cDNA synthesis. Following sequencing, the replacement of two A and G bands in the absence of CE with a sharper A band under CE treatment indicates a modification site. (**C**) In TRIBE‐seq and PUP‐2 RNA tagging, the protein of interest in fused to ADAR and PUP‐2 enzymes. ADAR converts adenosine to Inosine and PUP‐2 uridylates any RNA molecules that interact with the protein of interest. If all the RNA species inside the cell are sequenced, the A‐to‐I modified and uridylated RNAs indicate the ribonucleic acid partners of the protein of interest. (**D**) In BRIC‐seq, the cells are treated with 5′‐bromo‐uridine, and the decrease in labelled RNA levels is screened in real time.

Several methods have been developed for global or single‐molecule mapping of local primary and secondary structures of RNA molecules 56, 62. However, these methods are not efficient in revealing the long‐range tertiary structures and intermolecular RNA–RNA interactions 110. LIGR‐seq makes use of the modified psoralen derivative 4′‐aminomethyltrioxalen (AMT), which intercalates into double‐stranded RNA and joins them together upon irradiation at 365 nm 110. Ensuing cell lysis and partial digestion of single‐stranded RNA, the enriched duplexes are ligated to each other by circRNA ligase. The cross‐links are reversed by irradiation at 254 nm, and the RNA species are subjected to high‐throughput sequencing to reveal the chimeras. To correct for background‐ATM samples are used, and unligated samples are utilized to normalize for false chimeric RNAs 110 (Fig. 6A).

Previous attempts have been made to map miRNA–mRNA interactions in RNA induced silencing complexes 111 or snoRNA–rRNA interactions in spliceosome 112. However, all these methods require prior knowledge of the interacting protein partner for affinity purification and may lead to a high false‐positive detections due to *in vitro* cross‐linking 110.

Some lncRNAs are shown to function through interaction with other RNA molecules 113. A global snapshot of the RNA–RNA interactome achieved by LIGR‐seq releveled that at least one ncRNA is involved in the majority of stable interactions 110. Therefore, LIGR‐seq have the ability to reveal global maps of the lncRNA–RNA interactions, and shedding light on how perturbation of such interaction may lead to disease states including cancer (Table 1).

### Inosine chemical erasing sequencing (ICE‐seq)

Deamination of Adenosine to inosine is a common RNA modification, which plays important regulatory roles and has been implicated in several disorders including cancer 114. LncRNAs have been shown to undergo A‐to‐I editing with important effects on their secondary structures and interaction with other RNA species 13. As deregulation of such editing events may perturb lncRNA interaction networks and lead to disease conditions including cancer, a complete picture of normal or abnormal A‐to‐I editing is of paramount importance 115. Conventionally, sequencing and aligning cDNA molecules to their genomic loci can reveal such editing events 116. As Inosine base pairs with Cytosine, anywhere A has been replaced by G in the cDNA sequence information indicates an A‐to‐I conversion 116. However, this approach has several limitations including the necessity to exclude individual SNP variations and the inability to detect true I‐to‐G modifications from Gs occurring due to sequencing errors 116.

ICE‐seq makes use of Inosine cyanoethylation with Acrylonitrile. The resulting *N*1‐cyanoethylinosine adduct halts the cDNA extension process and allows identification of A‐to‐I editing points. The procedure is carried out with or without CE. In CE^−^ conditions both I and A positions recognized in sequencing. However, under CE conditions the RNAs undergoing A‐to‐I modifications stop to extend beyond the edition point. Therefore, the G band (I is read G in sequencing process) is erased, and a shaper A band is detected and that point is identified as a true modification site 116 (Fig. 6B). Although not yet applied to lncRNAs hitherto, ICE‐seq has the potential to reveal the deregulations that may occur in A‐to‐I editing of lncRNAs and how it may contribute to cancer (Table 1).

### Global mapping of lncRNA–protein interactions based on RNA modification and RNA‐seq

Despite being effective in determining RNA–protein interactions, RIP and CLIP can only unravel protein‐RNA interactions when specific antibodies against the protein of interest are already available 101. Two methods have been recently developed, which allow mapping of protein‐RNA interactions without the need to antibody affinity purification 117, 118. In one of the methods referred to as RNA tagging, global cataloguing of RNA–protein interactions is accomplished based on the ability *Caenorhabditis elegans* poly (U) polymerase (PUP‐2) 117. PUP‐2 does not possess any RNA‐binding domain and therefore cannot uridylate RNA independently. Any protein intended to be dissected regarding its universal interactions with RNA species inside the cell has to be fused with PUP‐2 protein. Then upon its interaction with any counterpart RNA, PUP‐2 uridylates the cognate RNA. The uridine‐tagged RNAs are finally enriched using U‐select primers and subjected to RNA sequencing, unravelling a global map of all RNAs interacting with the protein of interest 117. However, Uridylation using PUP‐2 can only reveal the interacting RNAs but not the exact RNA–protein interface 118 (Table 1). In another technique named targets of RNA‐binding proteins identified by editing (TRIBE), the protein of interest is fused to Drosophila RNA‐editing enzyme ADAR which converts adenosine to inosine 118. Therefore, any RNA that interacts with the protein of interest is subjected to A‐to‐I editing and reveals the approximate interaction interface after next‐generation sequencing (Fig. 6C). This technique, however, is not capable revealing the completely exact interaction site as in CLIP but circumvents the necessity to having a specific antibody against the protein whose interaction network is to be revealed 118. Unlike CLIP which is commonly carried out on tissues, TRIBE‐seq can be applied to a limited number of cells and be used to analyse RNA–protein interactions in homogenous cell populations (Table 1) 118.

These techniques have the potential to be extended to lncRNA research, shedding light on all the interactions a given protein of interest may have with the population of lncRNAs inside the cell and depicting probable interruptions in such interaction networks that may lead to cancer.

### Global run‐on assay sequencing (Gro‐seq)

A global understanding of genomic regions undergoing active transcription can provide valuable information for discovery and functional analysis of lncRNAs. Global run‐on assay sequencing (GRO‐seq) is a technique based on NGS technologies that provides valuable insights into of the location, orientation and density transcripts undergoing active transcription by RNA polymerase II. In this technique, the RNA polymerase is allowed to function in the presence the UTP analogue Bromodeoxyuridine (BrdU). The nascent RNAs are captured using anti‐BrdUTP antibodies and subjected to deep sequencing. The resulting sequence reads are aligned across the genome to map the genomic positions undergoing active transcription 119.

The technique, however, is limited to cell culture and other artificial systems due to its dependence on incorporation of Bromodeoxyuridine (BrdU). New initiation events that may happen and introduction of artefacts during preparation of nuclei are among other demerits of the technique 120 (Table 1).

Using this method, several studies have indicated that transcription by RNA pol II often occurs divergently and leads to production of lncRNAs 119, 121.

### 5′‐bromo‐uridine immunoprecipitation chase–deep sequencing (BRIC‐seq)

The result of many studies has shown that there is an intimate relationship between mRNA half‐life and its function, which implies an understanding the half‐life of lncRNAs can provide useful information about their functions 122, 123. Tani *et al*. 122, developed a technique named 5′‐bromo‐uridine immunoprecipitation chase–deep sequencing analysis (BRIC‐seq), to determine precise RNA half‐life inside the cell.

The method involves pulse labelling of cells with 5′‐bromo‐uridine and then quantifying the decrease in labelled RNA levels that occurs over time making use of deep sequencing. The result of this study indicated that RNA molecules with half‐lives <4 hrs include the majority of regulatory and lncRNAs 122 (Fig. 6D) (Table 1).

### Fluorescent *in situ* sequencing (FISSEQ)

RNA sequencing provides valuable insights into global quantitative and qualitative changes in transcripts but does not picture the spatial distribution of transcripts 124. On the other hand, *in situ* hybridization allows simultaneous visualization of cellular location of a small number of transcripts 124.

FISSEQ extends the applicability of RNA‐seq beyond *in vitro* quantitative measurement of transcripts to *in vivo* and allows spatial organization of gene expression to be revealed globally 125. In FISSEQ the cells are fixed, cDNA synthesis is carried out, and the cDNA molecules are circularized using CircLigase. To prevent diffusion, aminoallyl‐dUTP is used during reverse transcription and primary amines are joined together using PEG. Finally, SOLiD sequencing chemistry is employed to provide a quantitative spatial vista of gene expression 125.

FISSEQ has the potential to reveal the global spatial changes in lncRNA expression in disease conditions including cancer. However, the technique needs to be refined regarding its read length, sequencing depth and coverage and library preparation 125.

## Conclusion

The complexity of cells is better appreciated with this emerging view that most of the genome is functional and ubiquitously transcribed into a repertoire of coding and non‐coding RNAs. LncRNAs are highlighted in the new transcriptional landscape replete with transcripts of unknown function. They play vital roles in various cellular processes and disease conditions including cancer.. Therefore, considering their important regulatory roles, lncRNAs are among the missing parts of the puzzle in the long‐lasting fight against cancer and have to be scrutinized comprehensively so as to enhance our current understanding of this disease. The boundaries of our capability to understand how lncRNAs can contribute to cancer prevention or progression are contingent on power of the techniques available.

High‐throughput sequencing has accelerated the pace at which new lncRNAs are being discovered and allows global analysis of lncRNAs. However, our ability to characterize the expanding catalogue of unknown lncRNAs is also dependent on other techniques enabling us to map lncRNA interactions, their structure and function. For instance, the subcellular location of lncRNAs can be determined using FISH and further clarified by the combination of FISH and esi‐RNA technology (cKLAN). Compared to RNA FISH which can localize few transcripts in a single reaction, FISSEQ allows the research to be directed at quantitative global and spatial analyses of lncRNA expression. New technologies developed based on CRISPR‐Cas system can now be a complement to the current RNA visualization techniques as they may be better applied to non‐coding transcripts. Structural studies of lncRNAs have become more feasible using SHAPE. But SHAPE is limited to single‐molecule analyses carried out *in vitro*. To overcome the single‐molecule limitation of SHAPE, global structural analysis of the complete RNA reservoir or structurome has been now possible by PARS and Fraq‐seq. icSHAPE has extended the applicability of lncRNA structural studies by SHAPE to the realm of *in vivo*. LncRNA interaction networks have been clearly delineated using RNA‐centric approaches comprising ChIRP, CHART, RAP and protein‐centric methods including CLIP. dChIRP has enabled domain by domain interrogation of lncRNA interactions. CLIP has been refined regarding its throughput and resolution to iCLIP and PAR‐CLIP. Recently, further refinement of CLIP has led to irCLIP and eCLIP which allow more accurate and rapid determination of lncRNA–protein interactions. RNAi has allowed the knock‐down experiments employed in functional studies of lncRNAs and CRISPRi can increase the precision with which we can suppress lncRNA expression in reverse genetic investigations. TRIBE‐seq enables global mapping of lncRNA–RNA interaction network, without any pre‐existing antibody for affinity purification required in CLIP techniques. ICE‐seq allows the research to be directed at depicting a global picture of lncRNA epitranscriptome and is not confounded by individual variations due to SNPs. The half‐life of lncRNAs can be screened in real time using BRIC‐seq. GRO‐seq provides a picture of transcriptionally active genomic regions as it can capture nascent RNA molecules.

As aberrations in abundance, subcellular localization and interaction networks of lncRNAs are among key contributors to cancer progression, the technological toolkit described throughout this review is central to a deeper understanding of lncRNAs not yet characterized, revealing their likely widespread roles in cancer disease and opening new perspective for therapy.

## Conflicts of interest

The authors confirm that there are no conflicts of interest.
